# Stability challenges of carbon-supported Pt-nanoalloys as fuel cell oxygen reduction reaction electrocatalysts

**DOI:** 10.1039/d2cc05377b

**Published:** 2022-11-22

**Authors:** Tina Đukić, Luka Pavko, Primož Jovanovič, Nik Maselj, Matija Gatalo, Nejc Hodnik

**Affiliations:** Department of Materials Chemistry, National Institute of Chemistry Hajdrihova ulica 19 1001 Ljubljana Slovenia matija.gatalo@ki.si nejc.hodnik@ki.si; Faculty of Chemistry and Chemical Technology, University of Ljubljana Večna pot 113 1000 Ljubljana Slovenia; ReCatalyst d.o.o. Hajdrihova ulica 19 1001 Ljubljana Slovenia

## Abstract

Carbon-supported Pt-based nanoalloys (CSPtNs) as the oxygen reduction reaction (ORR) electrocatalysts are considered state-of-the-art electrocatalysts for use in proton exchange membrane fuel cells (PEMFCs). Although their ORR activity performance is already adequate to allow lowering of the Pt loading and thus commercialisation of the fuel cell technology, their stability remains an open challenge. In this Feature Article, the recent achievements and acquired knowledge on the degradation behaviour of these electrocatalysts are overviewed and discussed.

## Abstract 2

The chronological development of Pt-alloys as electrocatalysts and electrochemical characterisation methods in our laboratory.

## Graphical abstract

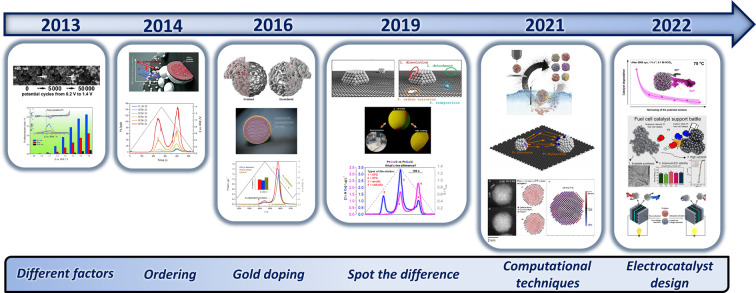

## 1. Introduction

An increase in greenhouse gas emissions and consequent climate change have pushed the establishment of a sustainable planet to the very top of global priorities. There is no doubt that the upcoming transition from conventional fossil-based to new renewable energy sources is essential.^1^ Among other approaches, a hydrogen-based future could be the main concept to achieve this.^2–4^ Namely, the hydrogen circular economy is based on the use of abundant solar and wind energy for producing hydrogen in electrolysers, while on the other hand, the fuel cells efficiently convert this hydrogen back to electrical energy. This way clean energy can be stored even for seasons. This could benefit all energy sectors, including transportation.^5^ When produced from renewable sources, hydrogen can be a zero-emission fuel for light-duty vehicles (LDVs) as well as a wide range of heavier applications such as heavy-duty vehicles (HDVs), buses, airplanes, ships, trains, *etc.*^6,7^

**Scheme 1:**
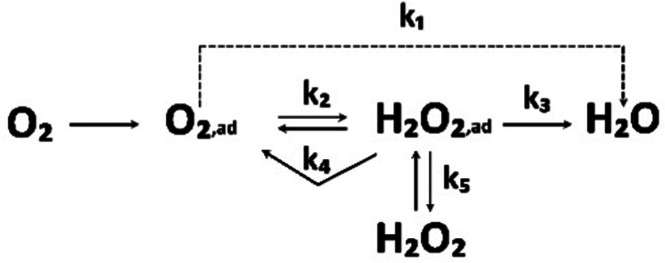
Simplified scheme of the basic ORR pathway. Reproduced from ref. 206 with permission from Elsevier, copyright 1976.

The most promising type of fuel cells to power the green transportation of the future are proton exchange membrane fuel cells (PEMFCs), based on a solid proton exchange membrane (PEM) that separates the anode and the cathode side in a so-called membrane electrode assembly (MEA).^8^ The process of converting hydrogen to electricity in the PEMFCs is conceptually quite simple. Supplied at the anode side of the PEMFC, hydrogen is oxidised and the formed protons travel through the membrane to the cathode side where they react with oxygen from the air creating electricity and water as the only by-product.^9^ However, these reactions need a catalyst, namely platinum (Pt) supported on the high-surface-area-carbons (HSACs) in the form of nanoparticles (NPs) (Pt/C). This nanocomposite was proven to be the best option for catalysis of both the anodic hydrogen oxidation reaction (HOR) as well as the cathodic oxygen reduction reaction (ORR).^10^ Pt is limited in accessibility and thus declared as a critical raw material (CRM). Both the scarcity of Pt as well as the consequent high cost of the catalyst material seems to be major obstacle to the massive scalability of PEMFC technology.^11,12^ Namely, the price of deficient Pt makes almost half of the total costs of the PEMFC system manufacturing^13^ even when considering the economies of scale.^14^ This is because unlike other components of the PEMFC, Pt does not benefit from increased production volumes.^14^ This is especially true for the cathode ORR electrocatalyst, since the required amount of Pt on the cathode due to the very sluggish kinetics is, in contrast to the anode, much higher.^10^ Therefore, the main motive in the development of PEMFC electrocatalysts is on one end to better understanding the mechanisms behind ORR (Scheme 1), while also optimising utilisation of Pt, in pursuance to get the highest conversion efficiency or power density with the least Pt.

To solve this major problem, researchers around the world are looking for new, highly active and low-priced ORR electrocatalysts. In addition to the Pt/C electrocatalyst, there exists 5 main groups of ORR electrocatalysts that are currently in various stages of development,^10^ ranging from laboratory validations performed mainly by using half-cell thin-film rotating disc electrode (TF-RDE) evaluation to small scale MEA testing and all the way to fuel cell stack-level testing. Namely, the groups are divided in de-alloyed (ordered or disordered) Pt-alloys,^15–19^ core–shell catalysts,^20,21^ non-precious group metal (non-PGM) catalysts,^22–28^ shape-controlled Pt-alloy catalysts^29,30^ as well as nanoframe Pt-alloy catalysts.^31^ What unites all of them is the aim to decrease or even eliminate (namely non-PGM electrocatalysts) the use of Pt and by replacing Pt/C electrocatalysts become the next-generation ORR electrocatalyst system to reach the production phase. Among the aforementioned groups, the de-alloyed Pt-alloys (or in other words carbon-supported Pt-based nanoalloys; CSPtNs) are so far the closest to achieve this goal.^10,32^ One of the reasons also might be the similarity to Pt/C and thus, also maturity of the production methods that enable synthesis of electrocatalysts with controlled Pt-alloy NPs size distributions. This includes namely various chemical or impregnation methods^33,34^ as well as some up and coming galvanic displacement methods.^16,35^ Consequently, CSPtNs will also be the focus of the present article. With CSPtNs the amount of Pt is reduced by the addition of less noble and at the same time non-critical metal (M). In other words, transition 3d metals such as Co, Cu, Fe and Ni are mainly alloyed with Pt in the core of the NPs (see pros and cons of different Pt-alloy systems in Table 1),^15^ thus, diluting Pt in the core and improving its utilisation on the surface, which results in higher electrochemically active surface area (ECSA; measured in units [m^2^ mg_Pt_^−1^]).^31,36–38^ At the same time, due to the combination of ligand, strain, coordination number and surface disorder effects, the intrinsic specific activity (SA; measured in units [mA cm_Pt_^−2^]) is enhanced.^39–46^ Consequently, the mass activity (MA; measured in units [A mg_Pt_^−1^]), the most evaluative industrial factor, is also increased.^47^ On the other hand, the utilisation of Pt is also improved by HSAC supports on which the Pt alloy is dispersed in the form of NPs. In addition, the carbon support also ensures high electric conductivity and adequate porosity of the electrocatalyst layer as well.^8^ The most common and widely used are ‘solid’ carbons such as Vulcan XC-72 as well as ‘porous’ carbons such as Ketjen Black EC300J.^48^ While ‘solid’ carbons enable good mass transport, Pt-based NPs suffer from ionomer poisoning and thus, inhibition of kinetic performance. On the other hand, ‘porous carbons’ have an opposite problem – the particles in the pores do not suffer from ionomer poisoning, however, particularly the smallest pores are poorly accessible for reactants, which has a negative effect on the mass transport. Consequently, the latest development moved in direction of highly graphitic mesoporous carbons with accessible pores that combine both the kinetic as well as mass transport advantages.^49^

**Table 1:** Advantages and remaining challenges of different Pt-alloys for the PEMFC application^18^

Alloy type	Advantages	Remaining challenges
Pt–Cu	• Facile formation of the intermetallic phase, which slows down the leaching of M	• Dissolved Cu blocks Pt surface
	• Very low carbon solubility in Cu (encapsulation of Pt–M NPs with a carbon shell is not a concern)	• The negative effect of dissolved Cu ions on the PEMFC performance – unacceptable degree for industrial application

Pt–Fe	• Facile formation of the intermetallic phase, which slows down the leaching of M	• Dissolved Fe ions act as strong Fenton reagent – increased PEM degradation
	• Fe is one of the most common elements	• The negative effect of dissolved Fe ions on the PEMFC performance – unacceptable degree for industrial application

Pt–Ni	• Much less detrimental effect of Ni dissolution on PEM degradation as Fe and Cu ions	• Difficult formation of the intermetallic phase (faster leaching of Ni)
	• Dissolved Ni does not block Pt surface	• Very high carbon solubility in Ni (encapsulation of Pt–M NPs with a carbon shell)
		• Improvements in the stability are still necessary for mass adoption by the industry

Pt–Co	• Facile formation of the intermetallic phase, which slows down the leaching of M	• Controversial mining practices (however, significantly lower amounts necessary per vehicle in respect to batteries)
	• Similarly to Ni, much less detrimental effect of Cu dissolution on PEM degradation as Fe and Cu ions	• Improvements in the stability are still necessary for mass adoption by the industry
	• Issues related to carbon solubility and encapsulation much less detrimental as in the case of Ni	
	• Dissolved Co does not block Pt surface	
	• Already applicable in the end-user products	

In addition to the high activity and low cost, long-term durability is also required to completely assemble the complex puzzle of an ORR electrocatalyst.^8,50^ However, while the activity of the state-of-the-art Pt-alloy electrocatalysts is already widely demonstrated and debated,^2^ much more still needs to be done to properly address their stability, which directly determines their long-term performance.^51^ Namely, the electrocatalyst can suffer from a wide range of degradation mechanisms: (i) electrochemically induced (transient) dissolution of Pt, which is closely related to the dynamics of formation/reduction of the Pt-oxide,^52^ resulting in Ostwald ripening^53,54^ and/or formation of metallic Pt bands in the membrane,^55–57^ (ii) dissolution of M^18,54^ and (iii) electrochemical and chemical carbon support corrosion,^58–60^ leading to the agglomeration and/or detachment of Pt NPs. All processes are interconnected (*e.g.* Pt catalyses carbon corrosion)^61^ and highly dependent not only on the intrinsic properties of the electrocatalyst such as choice of M,^18^ order/disorder,^17^ de-alloying/activation,^62^ type of carbon/degree of graphitisation,^63^ but on the operational conditions as well. Namely, recent research is providing significant evidence on the importance of the potential/voltage window as well as temperature on the degradation of not only carbon but also Pt in the case of CSPtNs. Additionally, clear connection has been established between the stability of Pt and the dissolution/leaching of M.^18,62,64^ On the other hand, while both metal dissolution and carbon corrosion are present in the more operational potential range of 0.6–1.0 V, metal dissolution nevertheless has a higher contributing. However, when the potentials/voltages exceed 1.0 V (*e.g.* during the start-up/shutdown), not only metal dissolution but also the kinetics of carbon oxidation reaction (COR), responsible for carbon corrosion, increases dramatically.^61^ Furthermore, recent research provides strong evidence that not only does carbon corrosion increases also with increasing temperature^58^ but so does the dissolution of Pt and consequently, also the dissolution of M.^54,62^ However, what is common with all the degradation mechanisms is that they lead to the loss of the active Pt surface area – ECSA, as well as a decline in the ORR mass activity. Furthermore, especially in the case of Pt-alloys, due to the additional dissolution of M in the PEMFC, not only the performance of the electrocatalyst itself but the overall performance and longevity of the PEMFC are affected. This is because the dissolution of M during PEMFC operation affects the proton conductivity, oxygen transport resistance, water uptake ability as well as speed of degradation of the membrane.^56,57,65^ Therefore, the understanding of degradation mechanisms at the level of electrocatalyst is of primary importance^18,66–68^ since their prevention directly affects the MEA level. However, in practice, the understanding of how electrocatalyst degradation affects the fuel cell operation is important for both theoretical and practical reasons since there are no catalysts that do not degrade over a prolonged time or upon exposure to extreme conditions.^8,18,54,56,58,62,69^

To bring the importance of stability of the ORR electrocatalysts closer to the scientific community, this feature article will focus on recent achievements in the domain of longevity of the CSPtNs. In the first part, general degradation mechanisms and their outcomes will be presented. Next, an up-to-date understanding of how different factors impact the stability of the state-of-the-art CSPtN electrocatalysts and the methods for their study will be provided. Ultimately, the review will provide recent knowledge on how to approach electrocatalyst design as well as PEMFC operation management to achieve high electrocatalyst performance and stability.

## 2. Degradation mechanisms of the carbon-supported Pt-nanoalloys

**Scheme 2:**
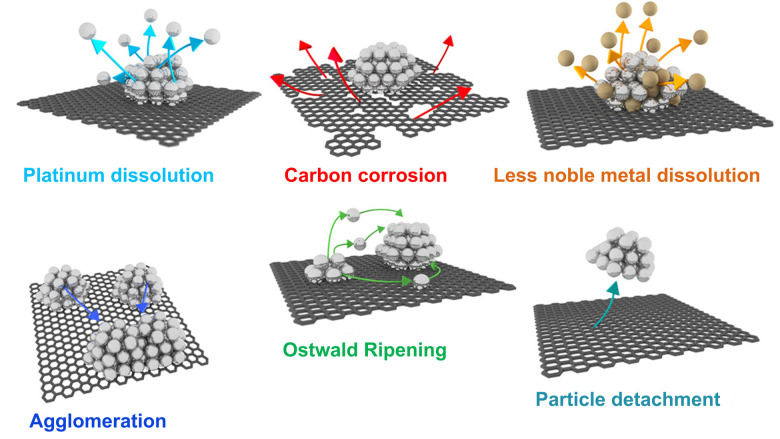
Schematic representation of primary (first row) and secondary (second row) degradation mechanisms of the state-of-the-art CSPtNs. Reproduced from ref. 207 with permission from American Chemical Society, copyright 2016.

Since the state-of-the-art CSPtN electrocatalysts are three-component nanocomposite systems including Pt–M NPs deposited on the HSAC support, the degradation of these electrocatalysts is a very complex phenomenon based on various degradation mechanisms (Scheme 2).^70^ Similarly to the pure-Pt NPs supported on HSACs, CSPtNs also primarily degrade due to carbon support degradation which manifests as carbon corrosion^57–59^ and degradation of Pt NPs which appears as Pt dissolution.^18,52,54,57^ However, in addition, Pt-nanoalloys also experience the dissolution of the M (dealloying or leaching).^18,54,64,71^ All of these mechanisms are interconnected and usually overlapped. Additionally, as a consequence of these principal degradation mechanisms, secondary degradation mechanisms also occur, such as Ostwald ripening,^53,54,57^ agglomeration^57,58,72,73^ and particle detachment.^74,75^ In the following text, each of the mentioned mechanisms will be described in more detail.

### 2.1 Metal dissolution

Although Pt is thermodynamically relatively stable in a wide pH range, it is nevertheless inclined to dissolve in a highly acidic environment (pH ≤ 1) and high (oxidising) voltages, typical for the PEMFC.^52,54,76^ In addition to these conditions, the dissolution of Pt is also dependent on the temperature,^54^ particles size,^77^ electrocatalyst loading,^78^ presence of the impurities (*e.g.* Cl^−^ ions),^79,80^ type of alloy,^18,54,64,81^ structure (ordering)^19,82,83^ and others.

**Fig. 1:**
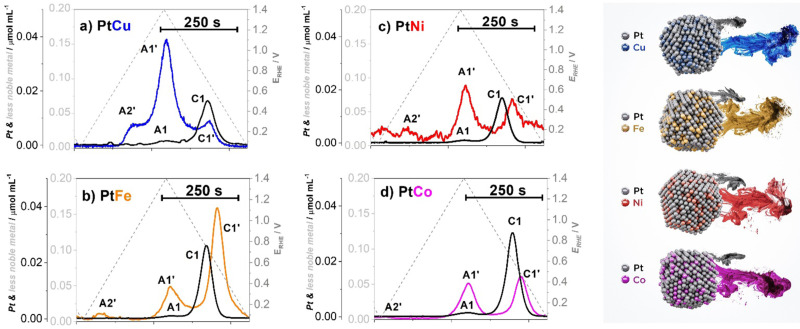
Metal dissolution of Pt–M/C electrocatalysts (M = Cu (blue), Ni (red), Fe (gold), Co (magenta)) with corresponding dissolution peaks (A1 and C1 – anodic and cathodic dissolution of Pt; A1′ and C1′ – anodic and cathodic dissolution of M; A2′ – additional anodic dissolution of M) measured using an electrochemical flow cell coupled to an inductively coupled plasma mass spectrometry (EFC-ICP-MS). Reproduced from ref. 18 with permission from iScience, copyright 2021.

Furthermore, it has been shown that whereas Pt is rather stable under (quasi)steady-state conditions (*e.g.* potential hold), the same does not hold true under transient conditions (*e.g.* potential cycling). This is known as a non-equilibrium or potentiodynamic dissolution, also referred to as transient dissolution. Under transient conditions, Pt can dissolve both anodically as well as cathodically.^52^ Anodic dissolution as part of the anodic potential or voltage sweep occurs during the oxidation of the metallic surface of the Pt-based NPs and thus, the formation of the Pt-oxide. This Pt-oxide begins to eventually penetrate the lower layers of crystal *via* the so-called oxide place exchange mechanism.^84^ While most of the surface Pt atoms get passivated by oxidation and thus, protected against any corrosion, penetration of the oxide into the lattice results in additional surface roughening and thus, the creation of dissolution-prone Pt defects and/or low-coordinated Pt sites. These defects/sites tend to get dissolved faster than they could be passivated (protected) by oxide formation (dissolution peak nominated as A1 – see Fig. 1).^52,84^ Whereas the upper potential/voltage limit (UPL/UVL) does have some influence on the extent of anodic dissolution^52^ (*i.e.* higher UPL/UVL, higher dissolution of Pt), the difference is rather negligible in contrast to the cathodic dissolution (dissolution peak nominated as C1 – see Fig. 1). The present understanding suggests that the extent of the oxide-place-exchange mechanism widely depends on the UPL/UVL. Namely, higher UPL/UVL results in deeper penetration of oxygen into the crystal structure of the Pt-based NP. During the cathodic sweep, the oxide is reduced, leaving behind a much higher amount of low-coordinated Pt sites which can ultimately dissolve. Therefore, in the case of Pt, cathodic dissolution has proven to be the dominant dissolution mechanism with respect to anodic dissolution.^84,85^

The dissolution of Pt further results in the secondary degradation mechanism.^53,54^ Namely, if dissolved Pt species from smaller particles redeposit back into larger particles, significant particle growth, known as Ostwald ripening, can occur.^53,54^ Thus, Ostwald ripening is a direct repercussion of Pt dissolution.^54^ In general, there are two types of Ostwald ripening: 3D Ostwald ripening when dissolved Pt species are travelling through the electrolyte, and 2D Ostwald ripening when dissolved Pt species are believed to diffuse along with the carbon support.^86,87^ Regardless of the type, it is worth considering that Ostwald ripening is also dependent on the inter-particle distance between NPs, since a larger concentration of dissolved Pt ions is to be expected when NPs are in close proximity to each other (in other words, in the case of high particle density regions).^70^ Thus, as a consequence of enhanced Ostwald ripening, a smaller inter-particle distance between NPs could also inhibit the possibility of Pt reaching the membrane and thus, formation of the Pt belt. Additionally, it has also been reported that ORR activity decreases with increasing inter-particle distance between NPs, which is especially true for smaller NPs at low metal loadings.^88^ Thus, in addition to stability, smaller inter-particle distance can also be beneficial for the ORR activity. On the other hand, if dissolved Pt NPs reach the membrane it causes the formation of the so-called Pt bands (accumulated Pt particles). These Pt bands accelerate polymer structure degradation of the membrane that results in the membrane thinning as well as can potentially form electronic short-circuits.^56,57^ In some cases Pt can also redeposit or get coordinated in the carbon support matrix and makes Pt single atoms sites.^89^

According to the Pourbaix diagrams, the M is in contrast to Pt thermodynamically unstable and is expected to dissolve already at very low potentials.^76^ Thus, when considering pure less noble metals, it is expected that less noble metals will experience complete dissolution during the first anodic potential sweep. However, in the event the M is alloyed with Pt, its dissolution becomes superimposed and thus mechanistically connected with the dissolution of Pt.^18,54,62,64^ This can be observed from the dissolution profile (Fig. 1) where it is clear that anodic dissolution of M (dissolution peak nominated as A1′) follows the anodic dissolution of Pt (A1), while the cathodic dissolution of M (dissolution peak nominated as C1′) follows a cathodic dissolution of Pt (C1).^64^ Furthermore, Pt alloys sometimes exhibit an additional M anodic dissolution (dissolution peak nominated as A2′) that is unrelated to Pt dissolution.^62^ As it will be explained in the continuation, while all Pt-alloys experience A2′ dissolution, the dominance of this peak is only very evident in the case of copper.^90–92^ Thus, in the case of Pt-nanoalloys, the dissolution of the M becomes largely dependent on one side properties of Pt itself, but also on the nature of the NPs.

When it comes to Pt-nanoalloys, dealloying or leaching of the M is not always an undesirable process. For instance, during the synthesis of CSPtNs, a critical step is related to the dealloying or in other words removal of the M from the at-most surface layers of Pt-based NPs to obtain a Pt-rich shell over the Pt-nanoalloy core.^64,93^ However, further continuous depletion/dissolution of the M, namely during the operation of the PEMFC, is an undesired and very detrimental process^69,94^ as it will always result in voltage losses and consequent decline in PEMFC performance.^62,95^ When it comes to the Pt-nanoalloy electrocatalysts, depletion of the M from the core of NPs leads to decay in ligand and/or strain effects, which in consequence results in a decrease in intrinsic ORR activity of the electrocatalyst.^57,96^ In specific cases when the chemical composition, as well as particle size, are above a certain critical threshold (*e.g.* M-rich as well as larger particles), dealloying can also lead to the formation of porosity.^17,97^ Moreover, the dissolved M ions can also strongly interact with the Pt surface and block the active Pt sites, thus inhibiting ORR.^98,99^ This is especially true in the case of copper dissolution since in the potential region of 0.45/0.6–0.7 V_RHE_ the dissolved Cu tends to redeposit back to the Pt surface in the form of up to one monolayer^90–92^ (known as copper underpotential deposition, Cu_UPD_^76^), which can be observed as an A2′ peak (Fig. 1a).^90,100^ Moreover, when it comes to the MEA, past research has shown that copper also can migrate from the cathode to the anode. Similarly, due to Cu_UPD_, the Pt surface of the Pt/C anode electrocatalyst gets blocked leading to a severe decrease in the HOR and thus, inadequate production of protons, which significantly lowers PEMFC performance.^90,101,102^ Less specific to copper, dissolved M ions are responsible for several further negative effects in the MEA that arise from their interactions with both the ionomer and the membrane. For instance, contaminants with such ions can reduce water uptake and form cross-links with sulfonate groups of the ionomer, which leads to an increase in tortuosity of the hydrophilic domain^69^ and consequently in an increased oxygen transport resistance.^69,103^ However, these effects are much less visible under “ideal” conditions (*e.g.* high RH and oxygen), whereas if studied under drier and warmer conditions (*e.g.* low RH and air) can be much more noticeable and better understood.^62,69^ Furthermore, the M ion contamination results in the formation of the cation-water clusters which have lower mobility than H^+^ clusters. This leads to decreased proton conductivity not only in the membrane itself but also in the ionomer thin film which surrounds electrocatalyst particles.^57,103,104^ Last but not least, the ions of dissolved M can chemically interact with PEM *via* the Fenton reaction. Namely, as the catalysts to the Fenton reagent, the cations of less noble metals catalyse the decomposition of hydrogen peroxide,^105^ which is to a certain extent always formed on a cathode in the two-electron pathway of ORR.^106^ Formed hydroxyl radicals further attack the polymer structure of the membrane ultimately leading to membrane degradation and thinning.^56,57,105,107^ It is worth mentioning that Pt can also act as a catalyst to the Fenton reagent but not so strong as the less noble metals since it catalyses the competitive reaction of hydroxyl radicals deactivation as well.^56^

### 2.2 Carbon corrosion

At the high potentials/voltages and/or high temperatures carbon is thermodynamically very unstable.^18,58,108^ Namely, at potentials above 0.207 V_NHE_ already at RT carbon is electrochemically oxidised by water to CO_2_,^76^ which is known as carbon corrosion.^58,61,108,109^ However, within the standard operating conditions of the PEMFC (*e.g.* voltage window from 0.6 to 0.9 V_RHE_) and without the presence of a catalyst, the kinetics of this process is very slow. Thus, realistically, under such conditions, the rate of carbon oxidation is relatively low.^110^ However, once both Pt that acts as a catalyst for not only ORR but also oxidation of the carbon, as well as elevated temperature, are added into the equation, the reaction rate is increased substantially, leading to more than negligible corrosion of carbon even in the operational potential range (0.6–0.9 V_RHE_).^58,61,109–111^ This catalysing effect of Pt in the carbon corrosion process is yet more pronounced when Pt-loading and ECSA are high.^112^ More importantly, in the case of the anode hydrogen–oxygen interface formation during the start-up/shutdown conditions and consequent fuel starvation, the voltage in the PEMFC can reach values as high as 1.6 V_RHE_ resulting in unprecedented levels of carbon corrosion. A similar problem can occur in the case of water flooding of the anode due to inadequate water management.^57,112,113^ In the case of Pt-nanoalloys, this is even more important as such high voltages are also incredibly damaging to the NPs themselves and result in high amounts of also M dissolution due to significant dissolution of Pt. Thus, such extreme voltage conditions should be avoided already at the stack management level of the PEMFC.^61^ Nevertheless, although the carbon corrosion is much more significant under start-up/shutdown and/or water flooding conditions, the effect of long-term operation must not be forgotten. Namely, the standard operation of PEMFC is much longer than the start-up/shutdown and water flooding processes. Hence, the cumulative impact of time of continuous operation of PEMFC on carbon corrosion is also present.^112^ This is especially significant for PEMFCs in HDVs, where operation time is expected to be up to 30 000 hours (in contrast to 5000 hours with LDVs).^114^ In any case, carbon corrosion is highly dependent on operational conditions (*i.e.* temperature and potential/voltage window),^58,108^ the properties of the carbon support (surface area, porosity, presence of functional groups, degree of graphitisation, *etc.*)^8,49,63,112,113,115,116^ and the type of support material.^8,14,63,113^

Carbon corrosion, the same as metal dissolution, results in several negative effects. Specifically, the excessively oxygenated functional groups generated on the carbon surface lead to the weakening of interaction between carbon support and Pt NPs, resulting in the secondary degradation mechanisms.^58,72–75^ Namely, the carbon support starts to shrink and lose its integrity. As a consequence, the neighbouring but initially separated Pt-based NPs located on this support start to migrate, which more often than not results in coalescence. This generates significant particle growth known as agglomeration.^58,72,73^ Ultimately, weakening of the interaction between metal particles and support due to carbon corrosion may cause even the detachment of the entire particles from the support.^74,75^ It is worth mentioning that coalescence and agglomeration can also occur without strong carbon corrosion, when the inter-particle distances are short, which is especially true for smaller NPs and smaller specific surface of the carbon support.^70^ Regardless to the source, both of them, agglomeration as well as particle detachment, result in lowering the electrocatalyst utilisation and decreasing the ECSA of the electrocatalyst.^57,58,70,112^ In addition, the electrochemical oxidation of carbon changes the chemical properties of the carbon surface, which results in increased hydrophilicity of the electrocatalyst support consequently leading to a decrease in oxygen permeability through the electrocatalyst layer.^57,112^ Moreover, structural changes caused by carbon corrosion are usually expressed by reduced porosity, which decreases electrocatalyst utilisation and causes oxygen transport limitations as well.^8,57,112^ This mass transport issue at the same time also impacts water transport through the membrane and increases water flooding effects.^72,112^ Lastly, on the catalyst layer level, carbon corrosion can also be recognised as thinning of the catalyst layer.^57,117^

## 3. Stability study approaches: the impact of different factors on the stability of Pt-based nanoalloy electrocatalysts

As described above, the complexity of the various degradation mechanisms of ORR electrocatalysts lies in their diversity as well as their dependence on different factors. Therefore, to study the stability of ORR electrocatalysts, it is necessary to apply methods that allow both: the study of (i) different degradation mechanisms as well as (ii) the dependence of these mechanisms on different factors. By obtaining such information, one can on one hand then design more stable electrocatalysts for real applications but also importantly, on the other hand, one can understand better how to operate and manage the PEMFC to extend its lifetime. Therefore, the focus of this part of the manuscript is on a combination of the latest methods for studying the durability of electrocatalysts by also simulating the real conditions in a PEMFC. In relation to this, also the effects of various factors on the stability of the CSPtNs will be discussed. This includes effects such as temperature, potential window, but also particle size, presence of impurities, structure of the NPs, supporting material type, *etc.*

### 3.1 Accelerated degradation tests (ADTs) in high-temperature disc electrode (HT-DE) setup for a closer to real electrocatalyst aging simulation

Due to the complexity and long duration of testing electrocatalysts in the MEA, the first step in the electrocatalyst evaluation is still the widely accepted laboratory-level TF-RDE methodology. In the combination with an ionomer such as Nafion®, the electrocatalyst is finely deposited on the electrode surface by drop-casting, creating a thin film. This method enables facile determination of ECSA, SA and MA. However, in order to avoid errors in the interpretation of the obtained data, the experimental parameters should be accurately set.^118^ The first step is the determination of ECSA, which can be calculated from the charge it takes to either adsorb/desorb a layer of hydrogen in the so-called hydrogen underpotential deposition region (H_UPD_) region in a cyclic voltammogram, or to oxidise a pre-adsorbed monolayer of CO in a stripping. For the correct determination of ECSA with both methods, a background correction for the carbon support must always be implemented. This step is very important since the ECSA is later used for the normalisation of the electrocatalytic activity, evaluated by measuring ORR polarisation curves. Thus, the SA should be calculated depending on the real surface area, based on the correction for purely geometric diffusion limitations. One such data treatment enables the determination of the catalytic activity independent of the electrocatalyst loading. Furthermore, the SA should be calculated from the kinetic current obtained *via* Koutecký–Levich equation at approximately half that of the diffusion-limited current, which should be determined at fixed potential of either 0.9 or 0.95 V_RHE_. Appropriately determined SA can be further used for the evaluation of the kinetic current per mass of electrocatalyst, *i.e.* MA.^118^

In addition to the determination of the intrinsic (kinetic) performance of the electrocatalyst,^118^ TF-RDE can be also useful for the stability studies of the electrocatalyst, by performing the so-called accelerated degradation tests (ADTs). Usually, ADTs are comprised of several thousands of electrochemical cycles in a specific potential window^61^ (*e.g.* 0.6–1 V_RHE_) using fast scan rates (*e.g.* 1 V s^−1^). However, while the usual US Department of Energy (DoE) guidelines for performing ADTs in a PEMFC are clearly in its usual operational range of up to 80 °C,^2^ most of the ADTs carried out in TF-RDE are still performed at room temperature (RT).^31,119–125^ On the other hand, only a handful of prior but very important studies include ADTs performed at elevated temperature^81,109,126,127^ and try to simulate this important parameter to get closer to the real PEMFC environment, thus, allowing for more precise analysis of electrocatalysts stability even during laboratory testing.

**Fig. 2:**
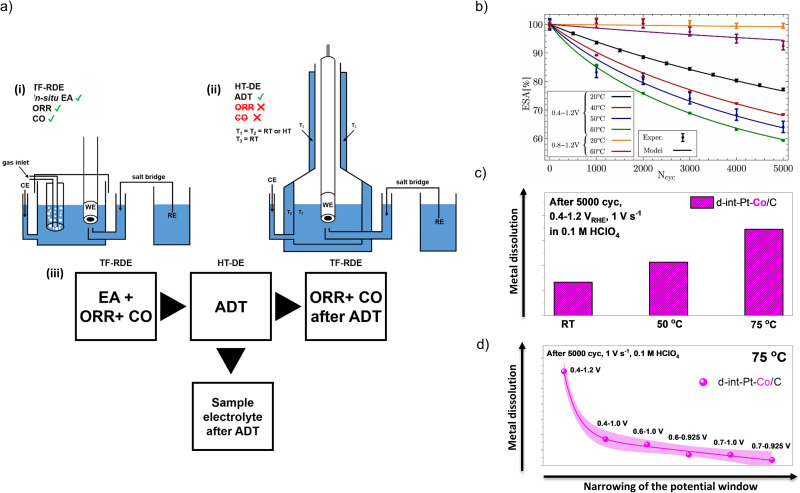
ADTs in HT-DE setup. (a) Schematic representation of (i) TF-RDE setup for ORR and CO electrooxidation measurements before and after ADT and (ii) HT-DE setup for ADTs at high temperatures; (iii) box-chart of the experimental flow used to evaluate the electrocatalysts at various potential windows and temperatures. Reproduced from ref. 54 with permission from American Chemical Society, copyright 2021, and from ref. 58 with permission from IOP Publishing, copyright 2020. (b) Comparison between experimentally measured and modelled changes in ECSA during different ADT protocols. Reproduced from ref. 108 with permission from Elsevier, copyright 2021. (c) Effect of ADT's temperature on the dissolution of M. Reproduced from ref. 54 with permission from American Chemical Society, copyright 2021. (d) Effect of ADT's potential window on the dissolution of M. Reproduced from ref. 54 with permission from American Chemical Society, copyright 2021.

Progress in this approach was recently demonstrated on Pt-based electrocatalysts, which were studied using a combination of the conventional TF-RDE and a so-called high-temperature disc electrode (HT-DE) setup, presented by our group.^54,58^ The setups allow for electrochemical evaluation of the electrocatalyst in the conventional TF-RDE setup before and after degradation, while ADTs are performed in a specially designed high-temperature cell, which can operate at up to 75 °C (Fig. 2a).^54,58^ While it has already been previously shown that the rate of corrosion of the carbon support follows the exponential Arrhenius law,^81^ mechanistic interpretations of the temperature-dependent kinetics of Pt dissolution remained highly speculative. Initial HT-DE data on benchmark Pt/C electrocatalysts (Fig. 2b) only revealed the correlation between loss of ECSA and choice of potential window (0.4–1.0, 0.4–1.2 or 0.8–1.2 V_RHE_) as well as the temperature (RT, 40, 50 or 60 °C), however, no clear conclusions on the possible effects of temperature on the dissolution of Pt could be made.^58^ To distinguish between the dissolution of Pt and corrosion of carbon, the data was then fed into a physical model (Fig. 2b) that provided some interesting findings.^108^ The results of the model suggest that an increase in Pt dissolution and consequential Ostwald ripening have a much higher contribution to an increase in the loss of ECSA at higher ADT temperatures than previously thought.

Our investigation was followed by studying intermetallic CSPtN electrocatalysts where HT-DE has been also combined with two *ex situ* methodologies, namely *ex situ* inductively coupled plasma mass spectrometry (ICP-MS) for determination of dissolved M and the *ex situ* transmission electron microscopy (TEM) for the visual observation of electrocatalyst degradation. Furthermore, our study also revealed a crucial significance of the ADT potential windows (from a rather narrow 0.7–0.925 V_RHE_ and up to a very wide and aggressive 0.4–1.2 V_RHE_; thus varying both the lower potential limits – LPLs, and upper potential limits – UPLs) as well as temperatures (up to 75 °C) on the loss of SA, MA, ECSA as well as loss of the M (Fig. 2c and d).^54^ The results have shown that a higher temperature of the ADT with a constant potential window (*e.g*. 0.4–1.2 V_RHE_) results in a larger loss of SA, MA, ECSA, but most surprisingly also a higher loss of the M (Fig. 2c). In addition, it has been shown that the studied CSPtN electrocatalyst experienced a linear increase in the degradation rate at 75 °C and ADT potential windows from 0.7–0.925 V_RHE_ and up to 0.4–1.0 V_RHE_ (Fig. 2d). In contrast, the degradation rate has been exponential when the UPL was further increased to 1.2 V_RHE_. This was once again true not only for SA, MA and ECSA but also for the loss of the M. Because the dissolution of the M from Pt-based nanoalloys is for the most part always a consequence of Pt dissolution,^64^ the results have hinted that a higher loss of the M with higher ADT temperature had to have occurred due to an increase in Pt dissolution. Thus, the indications of an increased Pt dissolution have been in line with the results from the prior model study^108^ on the Pt/C benchmarks. However, to finally resolve the so far speculative mechanistic interpretation of the temperature-dependent kinetics of Pt dissolution, the second half of this investigation focused on upgrading the electrochemical flow cell coupled to an inductively coupled plasma mass spectrometry (EFC-ICP-MS) methodology to enable not only time-and-potential resolved but also a temperature-dependent investigation of the metal dissolution from Pt-based nanoalloys presented in the next chapter.

### 3.2 Electrochemical flow cell coupled to an inductively coupled plasma mass spectrometry (EFC-ICP-MS) for *in situ* metal dissolution evaluation

To investigate the metal dissolution mechanisms, several variations of online detection inductively coupled plasma (ICP)-based techniques can be used.^128^ The use of ICP-based techniques in electrochemistry is already known for some time and is generally based on metal traces analysis. A breakthrough in the field was achieved by Ogle *et al.*^129^ with the establishment of the so-called atomic emission spectroelectrochemistry (AESEC), which in general is based on an online coupling of atomic emission spectroscopy (AES) with electrochemical analysis. The method made it possible to gain insight into the stability of various multielement, multiphase and composite systems by directly measuring their metal dissolution and evaluating their corrosion resistance.^129^ Later, variations of this method have been developed including a stationary electrochemical flow cell (EFC),^77^ a scanning flow cell (SFC)^128^ or a stationary probe rotating disc electrode (SPRDE)^130^ coupled to an ICP optical emission spectrometer (ICP-OES) or a mass spectrometer (ICP-MS).^128^

**Fig. 3:**
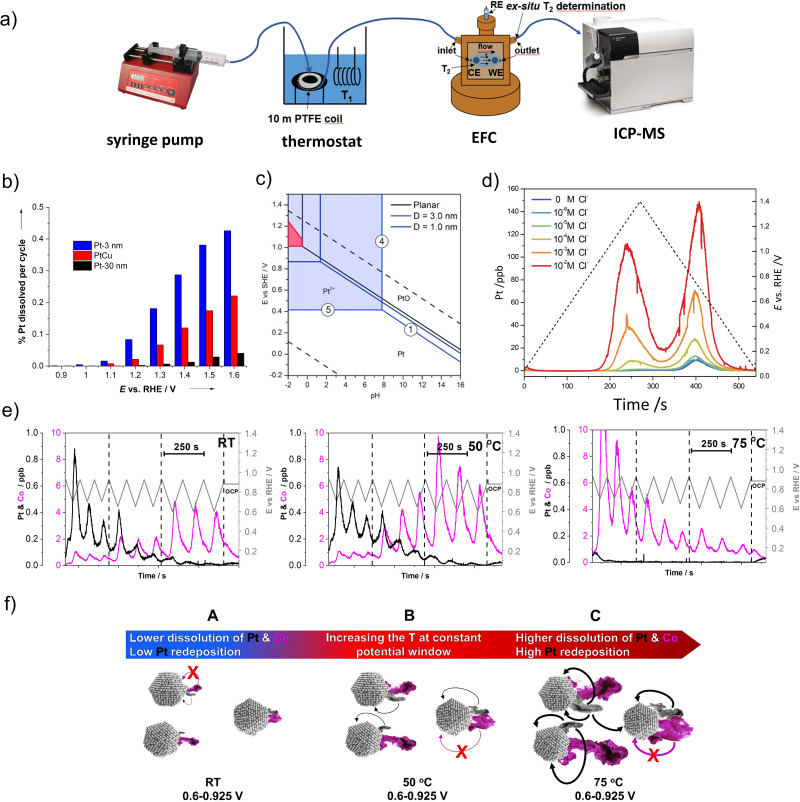
Metal dissolution measurements by EFC-ICP-MS. (a) Schematic representation of EFC-ICP-MS setup. Reproduced from ref. 54 with permission from American Chemical Society, copyright 2021. Effect of particles size on the Pt dissolution: (b) a mass percentage of dissolved Pt (from Pt-3 nm, PtCu and Pt-30 nm) per cycle plotted against the vertex potential of the cycle (reproduced from ref. 77 with permission from John Wiley and Sons, copyright 2013) and (c) a modified Pourbaix particle-size-dependent potential-pH diagram (reproduced from ref. 52 with permission from Elsevier, copyright 2016). (d) Effect of chloride impurities on the Pt dissolution: Pt dissolution profiles of the Pt/C during potential cycling (0.05 V–1.4 V_RHE_, 5 mV s^−1^), concerning different Cl^−^ concentrations (from 0 to 10^−2^ M). Reproduced from ref. 79 with permission from Royal Society of Chemistry, copyright 2014. (e) Effect of temperature and potential window on the metal dissolution: Pt and Co dissolution profiles of the d-int-Pt–Co/C during the LPL cycles (0.925−*X* V_RHE_; *X* = 0.7, 0.65, and 0.6; 5 mV s^−1^). Reproduced from ref. 54 with permission from American Chemical Society, copyright 2021. (f) Schematic representation of metal dissolution-related degradation mechanisms resulting from increasing the temperature at a constant potential window (black and magenta mists represent the dissolution of Pt and Co, respectively, whereas the arrows indicate the increasing presence of Pt redeposition). Reproduced from ref. 54 with permission from American Chemical Society, copyright 2021.

Since the SFC-ICP-MS and SPRDE techniques have already been widely addressed and described elsewhere,^128,130^ the present manuscript will focus on the EFC-ICP-MS methodology for time-and-potential resolved (*in situ*) determination of Pt-alloys dissolution, using a modified commercially available cell from BASi.^18,54,62,64,81,82,131^ The stationary EFC, which is coupled with an ICP (Fig. 3a),^77,132^ has a three-electrode system: working electrode (WE) and counter electrode (CE), which are two glassy carbon discs (*d* = 3 mm) embedded into polyether ether ketone (PEEK) material, and reference Ag/AgCl electrode (RE). The volume of the cell can be adjusted by a thickness of a silicon gasket used. On the WE, just like in the case of the rotating disc electrode (RDE), a thin film of an electrocatalyst can be applied. Since the surface of CE should be at least ten times larger than the surface of WE,^133^ appropriate amount of pure HSAC is deposited on the CE *via* drop casting. The discs are aligned in series so that the CE is placed first and the WE second in the direction of the electrolyte flow. The goal of such an arrangement of electrodes is to prevent the redeposition of dissolved metal ions from the catalyst layer on the WE to the CE, artificially lowering its detection in the mass spectrometer (MS). The flow system of the electrolyte to the cell is enabled by a syringe pump, while the cell outlet is directly connected to the ICP. From the syringe pump to the ICP, everything is connected with thin tubing made out of either polytetrafluoroethylene (PTFE) or PEEK.

One of the pioneering studies with this technique enabled the determination of the metal dissolution in both commercial carbon-supported Pt NPs with two different average particle sizes (3 and 30 nm) as well as in-house designed carbon-supported Pt–Cu NPs (5–50 nm) under a potentiodynamic regime by varying the UPL till up to 1.6 V_RHE_. The study has shown strong dependence on the cathodic dissolution of Pt with an increasing UPL. Dissolution of Pt has also shown a very strong particle size dependence (Fig. 3b).^77^ Namely, approximately seven times as much Pt is dissolved from 3 nm Pt particles as from 30 nm Pt particles. On the other hand, the PtCu alloy electrocatalyst with a broader particle size distribution (5–50 nm) exhibited considerably better stability than the 3 nm sample which was, however, still slightly lower than that of a commercial 30 nm Pt electrocatalyst. The results have been in line with the particle size effect and thus the tendency of smaller particles to dissolve already at low potentials, which is already known.^77,81,134^ This is also confirmed by a modified Pourbaix diagram for NPs of the diameter of 1 and 3 nm, discussed by Cherevko *et al.*^52^ From the diagram, which represents the particle-size-dependent potential-pH diagram (Fig. 3c), it is obvious that the equilibrium Pt/Pt^2+^ potential of 1 nm Pt NPs is significantly shifted downwards (at *ca.* 0.4 V_RHE_) in comparison with the 3 nm Pt NPs. Based on this analysis, it is believed that the dissolution of small particles (<4 nm) is predominantly an electrochemical process, while the dissolution of bulk Pt is more of a chemical process. Thus, particle size and distribution should be better controlled to obtain higher stability of the electrocatalyst.

Using the same technique, potentiodynamic Pt/C electrocatalyst corrosion has been studied as a function of chloride concentration. It was shown that chloride species not only enhance the corrosion rate but essentially change the corrosion mechanism when compared to a non-chloride environment. Namely, in the work of Pavlišič *et al.*,^79^ it has been noticed that the presence of chlorides lowers the Pt dissolution potential and increases the overall amount of dissolved Pt (Fig. 3d). Moreover, higher dependence of anodic dissolution on the presence of higher chloride concentrations was observed when compared to cathodic dissolution.

Furthermore, by using the highly sensitive EFC-ICP-MS system, the differences in the dissolution behaviour of the four most common CSPtN systems (Pt–Cu/C, Pt–Ni/C, Pt–Fe/C and Pt–Co/C) have been studied. Namely, the obtained EFC results on various Pt-alloys from Moriau and Hrnjić *et al.* have shown significant variations in the dissolution profiles of the M (Fig. 1).^18^ One of the most pronounced differences can be observed in the case of Pt–Cu alloy, which in addition to the anodic (A1′) and cathodic (C1′) dissolution of M also exhibits a distinct and pronounced underpotential deposition (UPD) peak (A2′).^76^ Due to this UPD interaction, Cu is, during the cathodic scan, partially redeposited back on the Pt surface, resulting in a relatively low-intensity C1′ peak in comparison to the other three investigated Pt alloys. On the other hand, this Cu is detected with a noticeable increase in the subsequent Cu_UPD_ dissolution peak (A2′) in the following cycle.^18,62,64^ In contrast, the other three Pt–M electrocatalytic systems do not experience relevant UPD dissolution. Despite that, a significantly less intense A2′ peak is nevertheless still present in the case of the other three alloys.^18,54,62^ However, the origin of this dissolution peak is not yet fully understood. Besides being UPD one speculation is that it could perhaps be related to the full reduction of Pt-oxide.^135^ Furthermore, while the Cu_UPD_ is the dominant dissolution mechanism at UPLs of 1.0 V_RHE_ as well as 1.2 V_RHE_, with higher UPLs such as 1.4 V_RHE_ anodic dissolution starts to become the prevailing dissolution process of Cu. In the potential window of 0.05–1.4 V_RHE_, a similar behaviour with anodic dissolution being the dominant dissolution mechanism has also been observed in the case of Pt–Ni/C.^18^ In contrast, in the same potential window, cathodic dissolution is the main dissolution mechanism for Pt–Co/C and Pt–Fe/C systems.^18,64^

As a continuation of the discussion related to the previous chapter (Fig. 2c), to finally resolve the so far speculative mechanistic interpretation of the temperature-dependent kinetics of Pt dissolution and to enable investigation of the metal dissolution from Pt-based nanoalloys at high temperature, EFC-ICP-MS has been recently upgraded with a thermostat (Fig. 3a) into a so-called high-temperature electrochemical flow cell coupled to an inductively coupled plasma mass spectrometry (HT-EFC-ICP-MS). As exemplified in the Pt–Co alloy (Fig. 3e), LPL cycles (3 cycles each LPL, 0.925−*X* V_RHE_; *X* = 0.7, 0.65, 0.6, 5 mV s^−1^, 0.1 M HClO_4_) at three different electrolyte temperatures (RT, 50 °C, and 75 °C) have been applied using HT-EFC-ICP-MS.^54^ While the LPL effect itself will be described separately in the following paragraph, the dissolution profile trends are very much dependent on the used electrolyte temperature. At first sight, Pt dissolution seems to decrease with increasing temperature, which is a trend similar to the observations made by Cherevko *et al.* on polycrystalline Pt.^136^ On the other hand, a significant increase in the less noble Co dissolution with increasing temperature is observed. More specifically, whereas Co dissolution is still relatively low at the highest LPL of 0.7 V_RHE_ in the case of the measurements performed at RT and 50 °C, it is already significant in the case of the measurement performed at 75 °C. To explain this, we need to consider two things: (i) to the prior work by Cherevko *et al.*, increasing temperature shifts the onset of Pt-oxide formation towards lower potentials and the onset of Pt-oxide reduction toward higher potentials.^136^ In other words, this means that at the same potential window (*e.g.* 0.6–0.925 V_RHE_) but with increasing electrolyte temperature, one not only forms more Pt-oxide anodically but also reduces more Pt-oxide cathodically, leading to a higher degree of predominantly oxide-place exchange induced dissolution of Pt. Since Pt ‘protects’ the M, the dissolution of Pt in the case of Pt-alloys is always followed by the dissolution of the M,^18,64^ thus, a higher degree of Pt dissolution also means more dissolved M; (ii) since we instead with the MS detector observe a decreasing trend (signal) for Pt dissolution with increasing temperature, this in some way contradicts our previous statement (a higher degree of Pt dissolution also means more dissolved Co). This could lead one towards an incorrect conclusion that perhaps Pt is not less stable but might be more stable when the increasing temperature. However, as already predicted as a possibility by Cherevko *et al.*,^136^ the observed decrease in the dissolution of Pt with increasing temperature is a consequence of the much more efficient redeposition of Pt. Thus, in reality, under a constant potential window (*e.g.* 0.6–0.925 V_RHE_), Pt dissolution and consequently also Co dissolution indeed both increase with increasing operating temperatures (Fig. 3f). At the same time, however, an even higher amount of dissolved Pt will redeposit back in the relatively thick (several μm) catalyst layers, most likely for the major part *via* the 3D Ostwald ripening mechanism,^137^ and thus unlike Co, does not reach the ICP-MS detector.^108^ This leads to the impression that we have dissolved less Pt rather than more. Thus, in the case of Pt-alloys, using the M can be considered as a probe, providing a rare opportunity to look beyond the limits of detection and connect the ‘invisible’ Pt signal with the ‘visible’ Co signal.

In addition to the temperature dependence, as part of the same experiment, the LPL effect was also evidently visible (Fig. 3e). More specifically, when applying a fixed UPL (in this case 0.925 V_RHE_) and upon decreasing the LPL from 0.7 to 0.65 and lastly to 0.6 V_RHE_, more Pt-oxide is reduced during the cathodic scan, which results in a higher amount of low-coordinated Pt-atoms. Due to the oxide-place exchange mechanism, these atoms tend to dissolve resulting in more uncovered (previously ‘protected’) M atoms and their higher dissolution.^54,62^ Thus, while avoiding high UPLs as a consequence of start-up/shutdown conditions is important mostly for avoiding severe carbon corrosion,^138^ it seems that the choice of LPL plays a different, but just as important role in extending the PEMFC lifetime. Moreover, looking at the present results, the role of the LPL might be of particular importance for the successful implementation of Pt-alloys in the PEMFCs.

Furthermore, we want to stress that not only temperature and potential window, but also other non-intrinsic factors influence the amount of dissolved and redeposited Pt. For example, in the case of a thicker electrocatalyst layer (*i.e.* by diluting a Pt/C electrocatalyst with a HSAC), the Pt dissolution remains the same but at the same time, the redeposition of Pt is increased, leading to a lower amount of detected Pt-dissolution. Thus, also a too thick catalyst layer, similarly as in the case of the temperature, can obscure the actual amount of dissolved Pt measured by ICP-MS.^116,139^

Last but not least, in addition to the metal dissolution measurements of Pt-based nanoalloys, it is worth mentioning that the EFC-ICP-MS technique is also applicable for stability studies of other metals such as gold,^140^ ruthenium,^132^ iridium,^141^ rhodium^142^ and palladium.^143^

### 3.3 Electrochemistry-mass spectrometry (EC-MS) for carbon corrosion studies

**Fig. 4:**
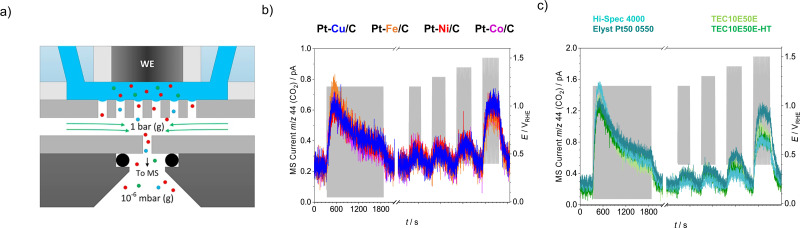
Carbon corrosion measurements by EC-MS. (a) Schematic representation of EC-MS (the cell-electrode assembly and membrane chip) in operation. Reproduced from ref. 151 with permission from Elsevier, copyright 2018. (b) CO_2g_ signals of different Pt–M/C electrocatalysts (M = Cu, Fe, Ni, Co). Reproduced from ref. 18 with permission from iScience, copyright 2021. (c) CO_2g_ signals of different Pt/C electrocatalysts (HI-Spec 4000, Elyst Pt50 0550, TEC10E50E, TEC10E50E-HT). Reproduced from ref. 18 with permission from iScience, copyright 2021.

Mass spectrometry can also be of significant relevance in the context of electrochemical stability of catalysts where it is regularly used to monitor gaseous products as markers for carbon support corrosion (*i.e.* COR), CO_2_ in particular. Typically, two variations of mass spectrometry-based techniques are employed for COR monitoring, differential electrochemical and online electrochemical mass spectrometry, DEMS or OLEMS respectively. Regardless of the configuration, the main principle of operation is based on sampling of the electrochemically evolved volatile species *via* a hydrophobic porous membrane and their subsequent transport to the MS detector enabling a time-resolved correlation of electrochemical and MS signal.^144,145^ Accordingly, mechanistic principles governing COR^110,146^ as well as stability trends of different carbon support analogues have been elucidated.^147–149^ In recent years such analytics has been additionally upgraded with the introduction of the so-called electrochemistry-mass spectrometry (EC-MS) technique developed by Chorkendorff's group and also sold commercially by SpectroInlets.^150,151^ Its principle of operation is based on a silicon membrane-based microchip functioning as a direct interface between a stagnant thin-layer electrochemical cell and a vacuum chamber (Fig. 4a), hence no differential pumping is needed. The chip is comprised of a sampling volume, which has a make-up gas flowing underneath carrying the volatiles into the mass spectrometer. A great benefit of this particular configuration is that all of the volatile molecules produced eventually reach the mass spectrometer, which on the high end enables product formation to be measured from total faradaic currents all the way down to 1 nA, corresponding to sub-monolayer sensitivity in sub-second time scale.^151^

In practice utilisation of the EC-MS technique has been most frequently demonstrated in the case of carbon corrosion monitoring where carbon-supported Pt (Pt/C) and carbon-supported Pt-based alloys (Pt–M/C) were investigated. In the study presented by Moriau and Hrnjić *et al.*^18^ the corrosion behaviour of the HSAC – so-called carbon black (Vulcan XC72), was investigated. The same support was used for all investigated Pt-based nanoalloys (Pt–Cu/C, Pt–Fe/C, Pt–Ni/C and Pt–Co/C). No significant differences in corrosion behaviour were observed (Fig. 4b), which indicates that the difference in Pt-alloying metal does not affect the corrosion of the carbon support. On the other hand, when compared with the Pt–M/C electrocatalyst (Fig. 4b), a notably higher signal of volatile CO_2_ was detected in the case of the Pt/C electrocatalyst (Fig. 4c). However, this phenomenon is attributed to the significantly higher loading for the Pt/C electrocatalyst (*ca.* 2–2.5 times higher).^18^

### 3.4 Modified floating electrode (MFE) setup for structure change studies

While the RDE method is undoubtedly efficient for the initial evaluation of the new electrocatalysts, their performance evaluation in the MEA setup often shows different trends,^2,29,32,152^ which should be ascribed to significantly different reaction conditions imposed by the two setups. Firstly, RDE testing is only applicable for low current density measurements,^118^ whereas a real PEMFC operates at several orders of magnitude higher current densities.^47,153^ Therefore, RDE data is typically only extrapolated to the high current densities, which frequently leads to erroneous predictions. Secondly, in contrast to RDE, MEA testing is significantly more complex, cost-intensive and time-consuming.^32,154^ Hence, the TF-RDE method is an appropriate technique for early-stage ORR electrocatalyst evaluation and screening.^62^ However, for a more meaningful performance prognosis, already at an early stage of catalyst development, electrochemical setups operating under real-like conditions need to be implemented. These would ideally withhold the simplicity of RDE, *i.e.* enabling accurate characterisation at high current densities while consuming only a small amount of catalyst. Indeed, few research groups have engaged in this direction implementing mainly two types of approaches, namely the so-called floating electrode technique (FET) and the gas diffusion electrode (GDE). Both approaches are designed in a way to allow supplying gas phase reactants directly to the WE. This enables obtaining high current densities because the mass transport limitations originating from limited solubility and slow diffusion of gases in liquids, which are typical in conventional setups like the RDE, can be avoided. The FET concept, introduced by Kucernak's group, requires a small amount of catalyst (with loadings as low as 0.16 μg Pt cm^−2^_geom_) enabling to perform electrochemical measurements of ultra-thin catalyst layers (down to 200 nm).^155^ These are deposited directly over a hydrophobic porous membrane (gold-coated polycarbonate track etched, PCTE), which enables to follow an intrinsic proceeding (*i.e.* without mass transport limitations) of a gas consuming reaction (HOR and ORR) under entire PEMFC-operation potential window. Accordingly, FET opened the path towards rapid and realistic studies elucidating several parameters such as break-in treatment, ionomer effect,^156^ Pt particle size effect and alloying effect for the case of Pt–Co analogues.^157^ Additionally, the absence of mass transport limitations offered a platform for kinetic modelling, which further contributed to the mechanistic understanding of ORR trends under realistic conditions.^157^ GDE setups on the other hand approach the conditions of PEMFC even further as they exploit the convective gas flow, a crucial parameter in circumventing mass transport limitations for larger electrode sizes (>1 cm^2^). Furthermore, the GDE cells can accurately measure the performance of realistic catalyst layers such as catalyst-coated membranes (CCMs).^158^ Overall, both the FET and GDE setups have proven themselves as powerful diagnostic tools capable of resolving either, detailed ORR mechanistic phenomena otherwise inaccessible with conventional setups (the FET case), or capturing the complex behaviour of a PEMFC catalyst layer (the GDE case).^159–161^ Accordingly, elucidation of rather poorly understood parameters can be pursued in a timely manner. Perhaps the most obvious diagnostic dimension lacking is the aspect of detailed structural characterisation of the catalyst layer. Namely, the structural properties of nanoparticulate electrocatalysts at the atomic scale are not stagnant but rather dynamic upon exposure to an electrochemical environment. These have a direct consequence on catalyst performance *via* the so-called structure–property relationships. Hence, the dynamics behind these changes might be crucial for the interpretation of ORR electrocatalyst activity and stability and such insights could significantly supplement the current understanding of electrocatalysts.

The application of these setups for detailed durability studies could be further supplemented by coupling them with other techniques such as identical location transmission electron microscopy (IL-TEM). The principle of the IL-TEM technique is previously reviewed and described.^66,68^ In general, it is used for local stability evaluation of the same spot of the sample during different stages of the electrochemical degradation protocol. The technique is based on a quasi*-in situ* repetitive process which includes three main steps: the sample observation by microscope before the degradation protocol, the degradation protocol, and finally the observation of the same spots after the degradation protocol. By observing several different NPs *via* this technique at different stages of degradation it is possible to track the exact history of the observed areas and get a deeper understanding of the structure-stability relationship of the electrocatalyst on an atomic level. Simulating IL-TEM results with computational methods such as Kinetic Monte Carlo (KMC) allows one to explain and better understand the processes in the obtained micrographic data.^66,68^ There is an example of utilisation of IL-TEM together with the KMC simulations, where it was shown that PtCu_3_ NPs corrosion is dependent on the type of facet since anisotropic facet dissolution of PtCu_3_ NPs proceeded in the order of {110} > {100} > {111}. Note that these investigations were performed in a conventional TF-RDE setup by attaching a TEM grid onto the glassy carbon RDE tip,^83^ hence further upgrade in understanding should pursue similar characterisation under a high current density regime.

**Fig. 5:**
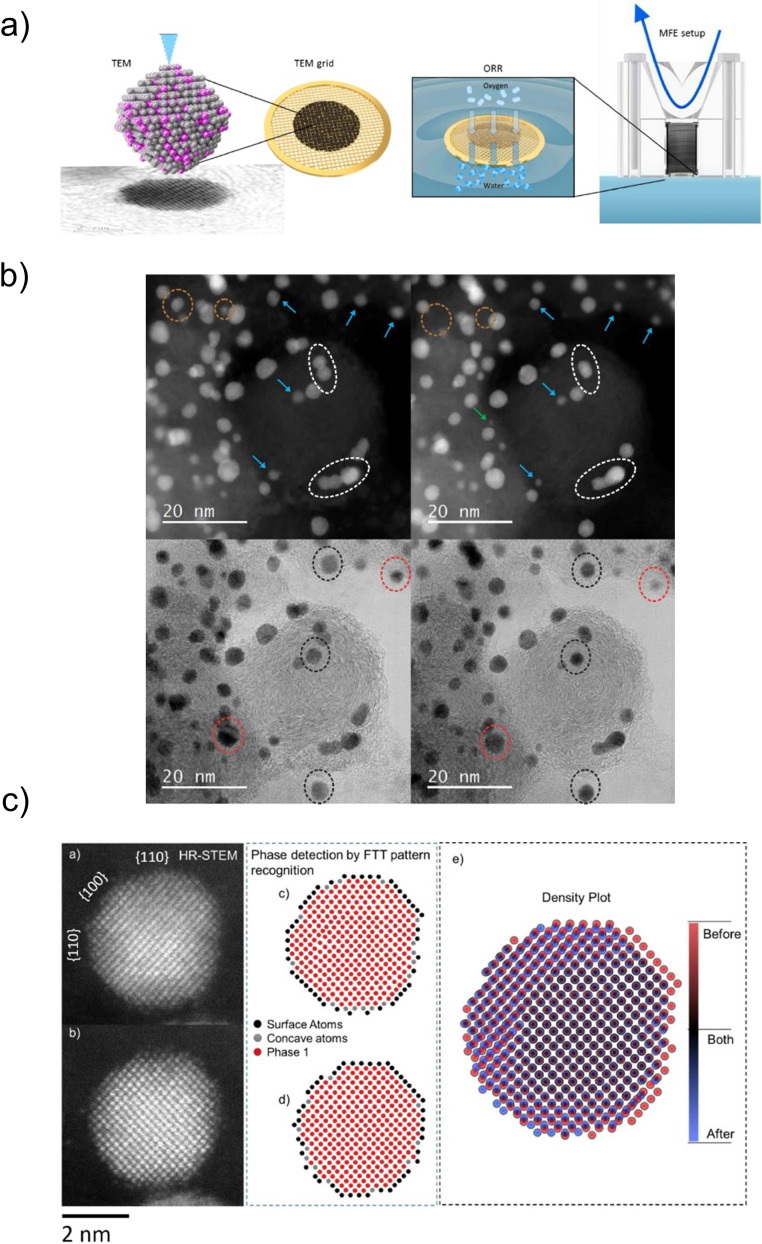
Structure change studies by MFE. (a) Schematic representation of the MFE setup. (b) Changes of the PtCo NPs. IL-TEM images of one region of the sample before (left) and after (right) electrochemical activation (200 cycles, 0.05–1.2 V_RHE_, 300 mV s^−1^): coalescence (white dashed circle), shrinkage and reshaping (blue arrow), particle detachment (green arrow, orange dashed circle), size reduction and Pt redistribution (red dashed circle), rotation and Pt redistribution (black dashed circle) and Co particle dissolution (lower right corner of the images). (c) Computer analysis by computer vision algorithm: IL-TEM images of Pt–Co NP (a) before and (b) after electrochemical activation (200 cycles, 0.05–1.2 V_RHE_, 300 mV s^−1^), atomic recognition images with phase detection by fast Fourier FFT pattern (c) before and (d) after, and (e) density plot of the atomic positions. Reproduced from ref. 67 with permission from Elsevier, copyright 2021.

Accordingly, we recently adopted the floating electrode concept by utilising carbon-coated TEM grids as electrode substrates (referred to from hereon as the modified floating electrode, MFE) (Fig. 5a).^162^ Although in this case the accessible current density range is narrower than in the case of GDE-based setups, the MFE still provides access to significantly larger ORR current densities than RDE (approaching the FET methodology).^163^ Similarly to FET, very small amounts of catalyst are needed (in the range of 5 μg_Pt_ cm^−2^_geom_) to perform the MFE measurement. Conveniently, electrochemical characterisation can be coupled with in-depth TEM analysis correlating electrochemical and structural insights. Our recent studies implemented this approach, particularly focusing on Pt-based alloys (Pt–M) in conjunction with the identical location approach (IL-TEM). This allowed us to follow individual structure-related events at the nano and atomic scale in between individual electrochemical perturbations.^67,163,164^ A careful analysis and comparison of atomically resolved high-resolution scanning TEM images of the same Pt–Co NP before and after MFE measurements revealed several ongoing processes: particle necking, anti-necking, pore formation, particle movement, coalescence, particle anisotropic etching and redeposition.^18,163^

With an eye toward easier, faster and more objective interpretation of the TEM images, an additional characterisation dimension of the modified floating electrode coupled to an identical location transmission electron microscopy (MFE-IL-TEM) approach has recently been introduced. Namely, the image analysis before and after MFE measurements utilises a new “*spot-the-difference*” microscopy image analysis algorithm,^68^ which enables extracting an atomic structure–stability relationship from the image. The approach was used to analyse specific facets of the Pt–Co NPs undergoing structural changes such as shrinkage (indicates dissolution), necking, anti-necking, detachment, particle movement, particle growth (due to agglomeration and coalescence), etching and/or redeposition (Fig. 5b and c).^66,67^ This characterisation approach enables deep insights into the restructuring of nanoelectrocatalysts at the atomic level.

## 4. Tuning the stability of Pt-based nanoalloy electrocatalysts

Understanding both the effects of PEMFC operation as well as the fundamental mechanisms for the degradation of Pt-based nanoalloy electrocatalysts presents a critical side of the coin to reaching longer PEMFC lifetimes. However, on the other hand, improving the intrinsic stability and thus, rational material design of novel and more durable electrocatalysts is just as important. In accordance to the different degradation mechanisms described in the Section 2, this part, thus, focuses on the recent progress towards tuning the Pt-based nanoalloy ORR electrocatalysts design and treatment conditions to stabilise their performance.

### 4.1 The Pt-alloy electrocatalyst design

**Fig. 6:**
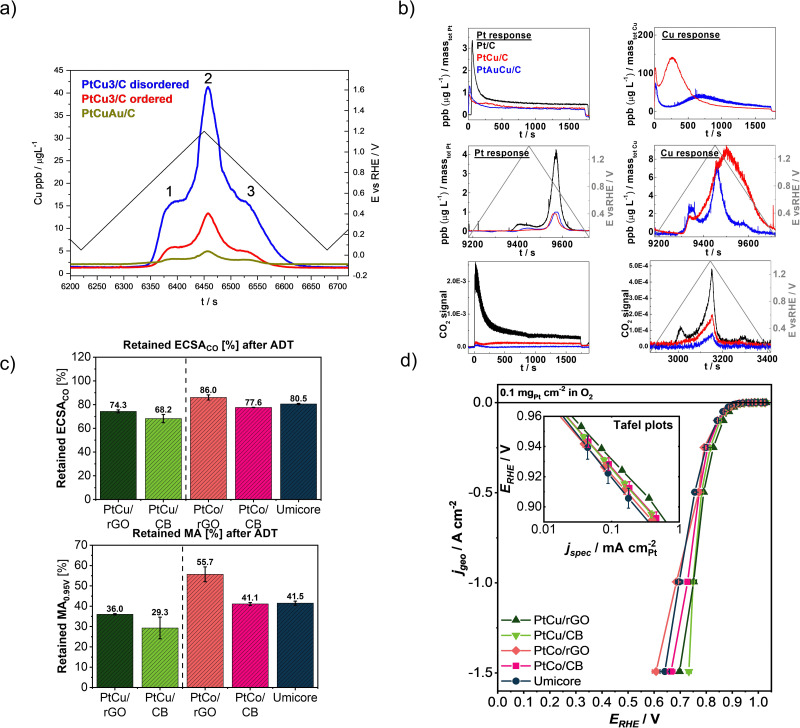
Improving the stability of the state-of-the-art carbon-supported Pt-based NPs by surface doping with gold: (a) Cu dissolution profiles of the PtCu_3_/C disordered, PtCu_3_/C ordered and PtCuAu/C during a potential cycle with the UPL of 1.2 V_RHE_ (reproduced from ref. 82 with permission from Elsevier, copyright 2016), and (b) Pt and Cu dissolution profiles (a–d) and CO_2(g)_ signals (e and f) regarding the applied potential window (reproduced from ref. 81 with permission from American Chemical Society, copyright 2016); by utilisation of rGO as support: (c) comparison of retained ECSA_CO_ and MA after ADT including 5000 cycles at 60 °C at 0.4–1.2 V_RHE_, 1 V s^−1^ in 0.1 M HClO_4_, and (d) ORR polarisation curves with inset figure showing Tafel plots, all measured in the GDE half-cell setup. Reproduced from ref. 63 with permission from American Chemical Society, copyright 2022.

Upon looking at the designing of a more stable CSPtN electrocatalyst there are two goals to reach: (i) a better stability of metals and (ii) a better stability of carbon support. Foremost, considering the extreme structural sensitivity of ORR as well as the complexity of the CSPtNs,^165^ optimising the structure is a key step in improving their activity and stability. Namely, there is clear evidence of the positive effect of structural ordering on the activity of CSPtNs.^17,166–168^ A mechanism is not completely clear but there are several explanations in the literature. One possibility is that the lateral strain in the Pt-skin of an ordered alloy is changed, thus the binding energies of adsorbates are changed as well.^39^ Another possibility is that dealloying from an ordered structure results in a skin type rather than skeleton type morphology and affects the ORR.^40,169,170^ On the other hand, the dependence of the electrochemical stability of M on the crystal ordering of NPs was also demonstrated. For instance, when comparing partially ordered PtCu_3_/C and its disordered temperature-treated analogue after electrochemical treatment (cycling either hold potential activation), the ordered sample exhibits better copper retention in both cases.^17^ The higher corrosion resistance of copper from the partially ordered structure is attributed to the higher average coordination of Pt with Cu atoms. Since the Cu–Cu bond is weaker than the Pt–Cu bond,^171^ means that the Cu dissolution is significantly increased in the case of a disordered structure (Fig. 6a).^17,82^ On the other hand, due to the greater coordination of Cu atoms, *i.e.* the higher amount of Pt–Cu bonds in the case of ordered structure, the intermetallic ordering enhances the Pt dissolution, which is nevertheless still very low.^82^

However, both Pt and M can be stabilised by doping with a small amount of a third metal such as gold.^81,82,172,173^ This was confirmed by doping the same ordered PtCu_3_/C electrocatalyst with slightly less than 1 atomic% of gold when highly unstable surface Cu atoms were galvanically displaced by gold and the PtCuAu was formed.^81,82^ By the already described EFC-ICP-MS method, metal dissolution profiles (Fig. 6a and b) were recorded for all three electrocatalysts: (1) structurally disordered *Fm*3*m* PtCu_3_/C, (2) structurally ordered *Pm*3*m* PtCu_3_/C, and (3) gold surface-doped PtCu_3_/C. In the Au-containing analogue, Cu dissolution was decreased by up to 60%. This stabilising effect of gold on the copper is attributed to the formed PtAu skin of NPs which better prevents Cu leaching and pore formation than a pure Pt surface. Furthermore, the presence of gold significantly decreases even Pt dissolution from Au-doped electrocatalyst, which is especially pronounced at potentials above 1.3 V_RHE_.^82^ The stabilising effect of gold on Pt stability is attributed to a shift in the oxidation potential of Pt to the more positive values, expanding the electrochemical stability potential window of Pt and inhibiting its oxidation/reduction.^81,82^ Moreover, the gold doping of Pt-based nanoalloy has a highly positive effect on carbon corrosion as well (Fig. 6b). Namely, when comparing the ordered PtCu_3_/C, its gold-doped analogue and a Pt/C standard, the lowest signal for CO_2_ was confirmed for the gold-doped sample (the carbon corrosion trend was the following: Pt/C standard ≫ ordered PtCu_3_/C > gold doped analogue of the ordered PtCu_3_/C). The lower oxophilic nature of gold is suggested to be the main reason for improved carbon support stability. Furthermore, both analogues of PtCu_3_/C electrocatalyst, but especially the Au-doped one, also exhibited a much higher Pt and carbon support stability than a widely used Pt/C standard.^81^ Furthermore, not only gold but also doping of Pt-nanoalloys systems with other noble metals such as Ru,^174^ Rh^175^ and Ir^176^ as well as other metals such as Mo^30,46,177^ can improve the stability of Pt-alloy NPs.

Some of the other highly relevant approaches to improve the durability of Pt-alloy NPs include the multimetallic core/interlayer/shell Pt-alloy NPs,^178^ shape-controlled octahedral Pt-alloy NPs,^30,179^ hollow/nano-frame Pt-alloy NPs,^46^ Pt-lanthanide alloy NPs,^46^ surface-confined Pt-alloy NPs,^46^ space-confined Pt-alloy NPs,^180–182^ different morphologies such as Pt-alloy nanowires, nanodendrites, *etc.*,^46,177^ and last but not least, also doping with non-metallic elements such as N,^183,184^ P and S.^46^

While the stability advancements of the CSPtNs metal phase focus mainly on the improving structure–property relationship of currently used alloys, there are two main development directions in the case of carbon supports.^8,113^ For instance, the first main development direction is focusing on improving the currently used high-surface-area carbon blacks (CBs). This is accomplished mainly by tailoring the CB support porosity type and size to achieve mesoporosity and improve the utilisation of the deposited Pt-NPs. The pores should be accessible, meaning there is an opening large enough to allow transport of gaseous species but also not too large to at the same time prevent the ionomer poisoning of the Pt-NPs located inside the pores.^49,185^ This approach can result in improved high current density performance and also improved durability of the catalyst.^49,185–187^ In addition, CBs can also be modified *via* doping with heteroatoms (*e.g.* N, B, P) where nitrogen emerged as the most promising candidate. By this, the Coulombic interaction between ionomer and N atoms in the carbon support is altered, which allows for a more even ionomer distribution during ink manufacturing process. This results in improved MEA performances, especially in the more relevant dry operating conditions.^27,188–190^ However, the second main development direction focuses on finding new, better alternatives to CBs. Since the first graphene isolation in 2004 by A. Geim and K. Novoselov,^191^ graphene and its derivatives emerged as a potential substitute for CBs as catalyst support.^192^ Graphene derivatives (GDs), in some form, can possess a higher specific surface area, better electronic conductivity and most importantly better thermodynamic stability than CBs.^8^ The latter should, if GDs get appropriately utilised as a support for ORR catalyst, provide significant improvement in increased resistance against carbon corrosion. However, more demanding properties of these materials such as higher hydrophobicity as well as their re-stacking tendency, make it difficult to achieve a high catalyst loading (*e.g.* >30 wt%) as well as its uniform distribution and thus sufficiently high ECSA. Both properties are of critical importance to achieve high roughness factors of the catalyst layer and, subsequently, sufficient high current density performance. Many new approaches on the synthesis part as well as at catalyst layer formulation are being proposed towards improving the properties of GD-supported materials, but none of them managed to match the performance of current CB-supported benchmarks. Recently Pavko *et al.*,^63^ proposed a unique, industrially scalable synthesis approach for the preparation of GD-supported catalysts based on a pulse-combustion reactor in combination with a double-passivation-galvanic-displacement method.^16^ With this innovative approach, highly homogeneous as well as a high metal-loaded Pt-alloy (up to 60 wt%) intermetallic catalysts on reduced graphene oxide (rGO), in this case, are achieved. The GD-supported catalysts were tested by HT-ADTs (described above) consisting of 5000 cycles in a potential window of 0.4–1.2 V_RHE_ at 60 °C. Enhanced durability is shown (Fig. 6c) when compared to the performance of CB (Ketjen Black EC300J) supported analogues as well as a commercial benchmark (Elyst Pt30 0690). Additionally, in combination with Raman spectroscopy and X-ray photoelectron spectroscopy (XPS) Auger characterisation, a clear connection between sp^2^ content and structural defects in carbon material with the catalyst durability is observed. A lower *I*_D_/*I*_G_ ratio, *i.e.* higher graphitisation degree and fewer structural defects (higher sp^2^ content), results in increased carbon support durability which boosts the overall stability of the catalyst composite. To investigate the high current density performance, the advanced GDE was used (Fig. 6d). Results show that the GD-supported catalysts exhibit excellent mass activities and possess the properties necessary to reach high currents if utilised correctly. Record-high peak power densities in comparison to the prior best literature on Pt-based GD-supported materials are achieved, which is promising information for future application.^63^

### 4.2 Additional treatment of Pt-alloy electrocatalysts – dealloying

**Fig. 7:**
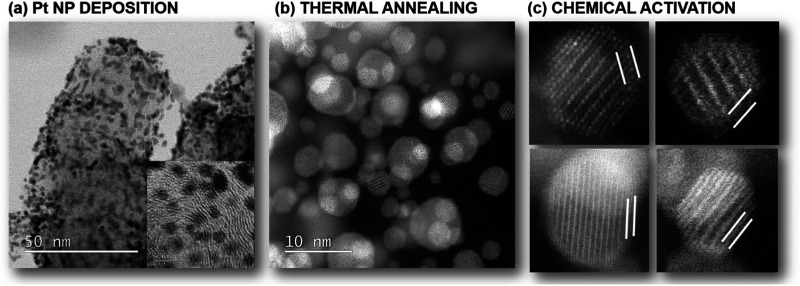
The three-step process for the preparation of state-of-the-art carbon-supported Pt–M electrocatalysts: (a) Pt NPs deposition, (b) thermal annealing, (c) chemical activation. Reproduced from ref. 62 with permission from American Chemical Society, copyright 2022.

By now it is clear that both the electrocatalyst performance and the durability are highly dependent on the structure of Pt-alloy NPs as well as the properties of the carbon support. While tailoring the bulk (intermetallic) crystal structure^15,17,183,184,193^ or controlling the shape^30,179^ of CSPtNs has been shown as highly effective, the importance of CSPtNs electrocatalyst activation is also highly essential.^62,93^ Generally speaking, activation refers to the transition of the electrocatalyst from an inactive to an active form. When talking specifically about CSPtNs, the electrocatalyst can be inactive or experience sub-optimal performance due to several reasons, however, we can ultimately group them into two major groups: (i) coverage of the active Pt-surface with a thick carbon shell and/or leftover organic molecules (*i.e.* surfactants) as residue from the synthesis^194^ and (ii) less noble metal (M) impurities.^195^ The latter and its relevance to CSPtNs will be the focus of the present discussion. Typically, one has to deposit Pt in the form of NPs on a carbon-based substrate (Fig. 7a). If such a composite is thermally annealed in presence of M, one can obtain Pt-alloy NPs (Fig. 7b) which are usually fully mixed with a substantial amount of M present at the top-most layers of the NPs. Since typical M are poorly active for ORR as well as thermodynamically highly unstable in acidic media, one has to transition CSPtNs from an inactive to an active state. This is namely achieved by electrochemical dealloying, enabled by potential cycling either potentiostatic conditions,^64,196,197^ or chemical dealloying which in general is based on acid washing.^62,93,197,198^ In both cases, the M is depleted from the NP shell and a Pt-rich overlayer is formed (see Fig. 7c). As suggested by the name, the remaining Pt-‘rich’ surface structure is not a classical ‘Pt-skin’ structure, but rather still retains a small amount of M.^93^ While the amount of M in the Pt-rich overlay is low in contrast to the amount present in the Pt–M core, it is nevertheless of significant importance to adequately activate a CSPtNs electrocatalyst.

**Fig. 8:**
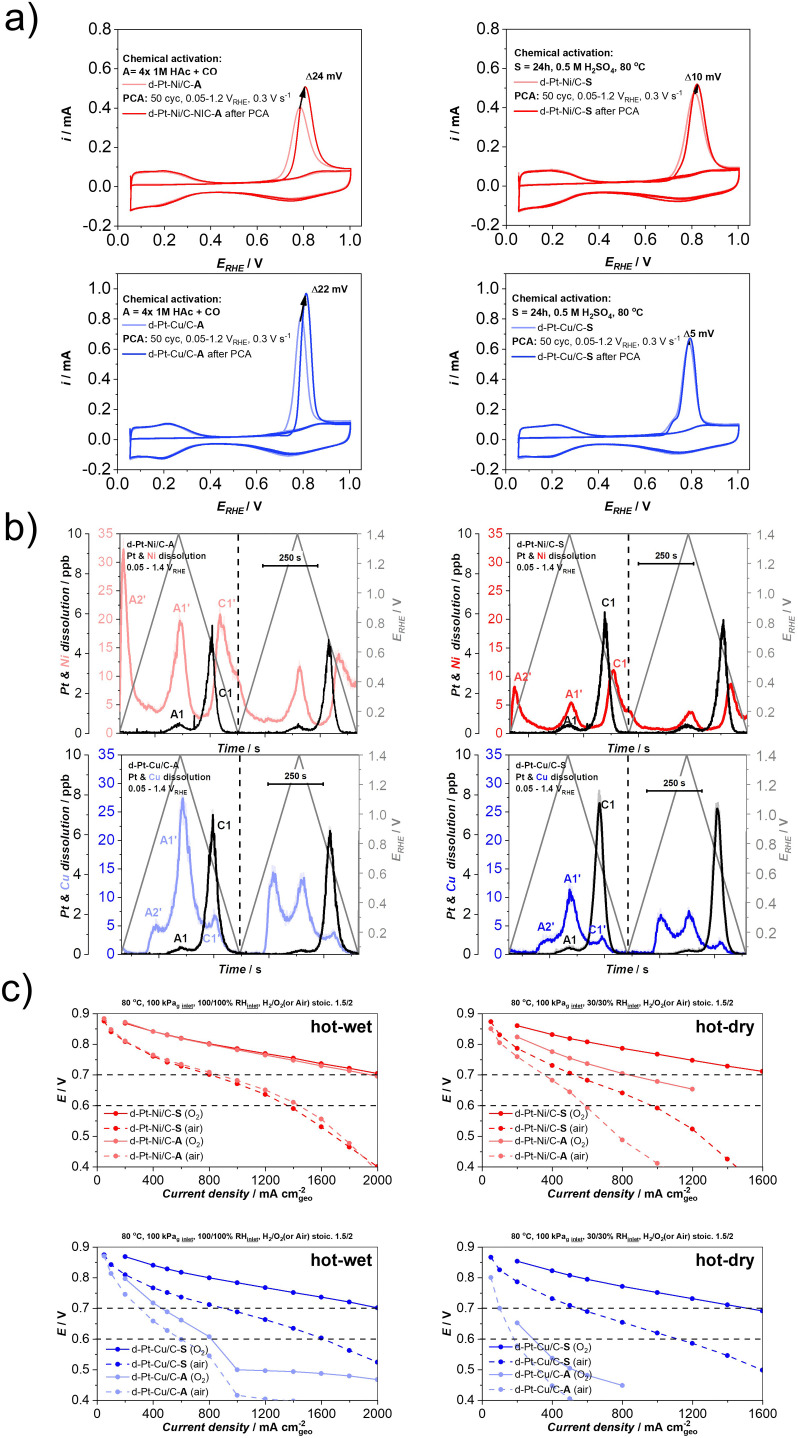
Improving the stability of the state-of-the-art carbon-supported Pt-based NPs using the appropriate activation method. (a) CO-electrooxidation comparison measured with TF-RDE for A- (d-Pt–Ni/C-A – red and d-Pt–Cu/C-A – blue) and S-activated (d-Pt–Ni/C–S – red and d-Pt–Cu/C–S – blue) electrocatalysts, before and after an additional 50 cycles of PCA (0.1 M HClO_4_, 0.05–1.2 V_RHE_, 300 mV s^−1^). (b) Comparison of metal dissolution from A- and S-activated electrocatalysts during cycling between 0.05 and 1.4 V_RHE_. (c) Comparison of ORR polarisation curves for A- and S-activated Pt–Ni/C and Pt–Cu/C at hot-wet and hot-dry conditions of the MEA. Reproduced from ref. 62 with permission from American Chemical Society, copyright 2022.

While the varying electrochemical dealloying conditions, such as scan rate, UPL, scan time, and the number of cycles, can primarily improve ORR activity,^196^ the chemical activation can be a convenient approach to improve electrocatalyst stability. To showcase the importance of adequate activation, Gatalo *et al.*^62^ used different two chemical activation protocols for the activation of CSPtNs, namely comparing a milder acetic acid activation^62,93^ as well as a stronger sulphuric acid activation for activation of Pt–Cu and Pt–Ni alloys.^199–201^ This resulted in evaluation of both A- and S-activated analogues of Pt–Cu/C (Pt–Cu/C–A and Pt–Cu/C–S, respectively) as well as A- and S-activated analogues of Pt–Ni/C (Pt–Ni/C–A and Pt–Ni/C–S, respectively) electrocatalyst. The liquid half-cell TF-RDE testing was used as a primary, fast and facile method for evaluating the analogues. All electrocatalysts were evaluated by measuring the ORR, followed by CO-electrooxidation, before and after additional dealloying initiated *via* potential cycling activation (PCA; 50 cycles, 0.05–1.2 V_RHE_, 300 mV s^−1^). Whereas the ORR polarisation curves before and after PCA overlapped when comparing the A- and S-activated analogues, both A-activated analogues (Pt–Cu/C–A as well as Pt–Ni/C–A) exhibited a visibly larger potential shift in the peak maximum, corresponding to CO-electrooxidation, than their S-activated analogues (Pt–Cu/C–S and Pt–Ni/C–S) (Fig. 8a). This was the first clue indicating that the A- and the S-analogues might be different, however, one could not consider just how significant the difference was without further inspection. In continuation, all 4 analogues were further investigated for metal dissolution by using the already thoroughly described EFC-ICP-MS method. By exposing the electrocatalysts to two cycles at 0.05–1.4 V_RHE_ to initiate significant dissolution, both A-activated electrocatalysts (Pt–Cu/C–A as well as Pt–Ni/C–A) exhibited noticeably higher dissolution of M than their S-activated analogues (Pt–Cu/C–S and Pt–Ni/C–S) (Fig. 8b) – confirming yet another difference between the A- and S-activated analogues. Lastly, the ORR polarisation curves evaluated in 50 cm^2^ MEAs at Johnson Matthey were compared for both A- and S-activated electrocatalysts in both O_2_ and air, as well as at both hot-wet (80 °C, 100% of relative humidity) and hot-dry (80 °C, 30% of relative humidity) conditions (Fig. 8c). This enabled simulating both more “ideal” (*e.g.* in the case of high relative humidity and/or use of oxygen) as well as more “realistic” (*e.g.* in the case of low relative humidity and air) conditions in an MEA. Thus, what initially started as a ‘simple’ shift in CO-electrooxidation peak maximum in the case of A-activated analogues (Fig. 8a), further revealed also as higher dissolution of M (Fig. 8b) and lastly translated in significantly inferior performance in an MEA (Fig. 8c). Namely, this difference in performance was most significant when comparing A- and S-activated analogues under more realistic hot-dry conditions in air. This suggested that a milder chemical activation leads to a less stable Pt-alloy that experiences a higher amount of M dissolution, which can already be detected using the previously described TF-RDE protocol *via* the shift in CO-electrooxidation peak maximum. This lower stability against M dissolution ultimately results in a higher presence of M impurities in the catalyst layer and the membrane of an MEA, most likely impacting various mass transport-related parameters such as proton conductivity, oxygen transport, water uptake, *etc.*^69,103^ Ultimately, the results show that the same Pt-alloy electrocatalyst can experience many fundamental differences, which are highly dependent on the choice of the dealloying protocol, emphasising the importance of the electrocatalyst activation protocol and applied conditions.

## 5. Concluding outlook and future trends

While hydrogen is expected to be the key to establishing a fossil-free society, one of the bottlenecks in maturing its technologies like proton exchange membrane fuel cell (PEMFC) is still oxygen reduction reaction (ORR) electrocatalyst. The state-of-the-art carbon-supported Pt-based nanoalloys (CSPtNs) show the greatest potential to overcome this obstacle. However, the research must be concerned not only with the activity and costs but, maybe even more importantly, with the long-term durability as well. The methods of investigation of complex degradation mechanisms of the CSPtNs are the main tool to achieve it. Although the commonly used thin-film rotating disc electrode (TF-RDE) method, as well as advanced methods such as electrochemical flow cell coupled to an inductively coupled plasma mass spectrometry (EFC-ICP-MS) or electrochemistry-mass spectrometry (EC-MS), can promise extremely valuable information in the stability estimation of the new electrocatalysts,^18,54,62^ utilisation of such methods alone is not enough for a profound understanding of the electrocatalyst behaviour in a real fuel cell. The method that can help for resolving this issue is for sure the modified floating electrode (MFE) approach, which provides both: (1) an understanding of the local electrocatalyst behaviour at the atomic scale, and (2) insight into the electrocatalyst durability at high current densities.^67^ However, we believe that only by applying all these methods together, one can get a quite fast and at the same time comprehensive perspective on the ORR electrocatalyst stability at the laboratory scale. Still, further methods development will be a huge benefit to the structure–function understanding of Pt-alloy electrocatalysts.

On the other hand, the development of new electrocatalysts has to be focused on searching for the perfect structure, composition of metals and the support. In this regard, novel supports (such as graphene) and ternary Pt-based nanoalloys could play an important role.^63,81,82^ Additionally, the establishment of a standard protocol of activation can also be beneficial.^62^ Nevertheless, although a lot of achievements have already been made in the field of electrocatalyst stability, the challenges in the stability of the state-of-the-art CSPtNs now are moving from the *ex situ* to the *in situ* level, where a lot remains to be resolved. First of all, stronger relationships between research associations, industry and governments are indispensable to enable state-of-the-art CSPtNs to break into the market and meet society's requirements. Namely, for Pt-alloys to reach the production phase and replace the conventional Pt/C electrocatalysts,^10^ Pt-alloys must meet the harsh stability requirements as well as other technical targets imposed by likes of Clean Hydrogen Joint Undertaking in Europe,^202^ Department of Energy in the USA^203^ as well as New Energy and Industrial Development Organisation in Japan.^204^ However, this will not only be achieved by intrinsic advancements in electrocatalyst stability but also in the advancements in understanding the PEMFC operation and thus, also the non-intrinsic impacts governing electrocatalyst stability. As described in the previous chapters, metal dissolution, as well as carbon corrosion, are highly dependent on the voltage window at which the fuel cell operates. For instance, avoiding start/stop conditions that result in exceptionally high upper voltages is critical not only due to triggering of the massive amounts of carbon corrosion (and consequent coalescence/agglomeration of Pt-based nanoparticles (NPs))^58^ but also substantial dissolution of Pt. In the case of Pt-alloy NPs, however, this also leads to the consequential dissolution of M as a result of additional dealloying.^54^ Furthermore, it has been also shown that one can additionally limit the dissolution of M by firstly controlling the upper voltage limit (UVL) as low as possible (*e.g.* even below 0.925 V_RHE_), but at the same time also limiting the lower voltage limit (LVL) (ideally above 0.6 V_RHE_).^54,62^ Thus, from the engineering perspective, it is vital when using Pt-alloy cathodes to aim at catalyst layer formulations with close to maximum current densities at already relatively high operating voltages. The reasoning behind the avoidance of particular UVLs is namely to avoid excessive oxidation of the Pt surface of Pt-alloy NPs and limit the extent of the oxide-place exchange mechanism.^52^ This is because the oxide-place exchange mechanism ultimately results in (significant) cathodic dissolution of Pt (*e.g.* during vehicle acceleration when high power is required and the cell voltage drops). In the case of Pt-alloys, cathodic Pt-dissolution is always consequently followed by additional dissolution of M. And exactly since M dissolution is in fact a consequence of Pt dissolution,^18,54,62,64^ it is critical to shift the focus away from merely trying to solve the stability of Pt-alloys and additionally focus on how to get around the limitations of the intrinsic stability of Pt with just one example being the necessity of limiting the voltage window to a very narrow but a very efficient one.^54,62,205^ In other words, it is necessary to define an optimal voltage window while maintaining a compromise between the highest current densities and the smallest impact on the stability of the CSPtN electrocatalysts, thus, preventing any major Pt dissolution to occur in the first place. Furthermore, the interaction between the ORR electrocatalyst and other PEMFC components must be addressed to achieve a stable assembly.^8,56,57^ Thus, also advancements such as for instance highly-accessible (mesoporous) supports as well as high-oxygen permeable ionomers are in this aspect highly synergistic to achieve such stable assemblies as they ultimately also enable enhancements in current densities above 0.6 V.

## Author contributions

Tina Đukić: Conceptualisation, writing – original draft, writing – review and editing, visualisation. Luka Pavko: Resources, writing – review and editing. Primož Jovanovič: Resources, writing – review and editing. Nik Maselj: Writing – review and editing. Matija Gatalo: Resources, writing – review and editing, supervision. Nejc Hodnik: Conceptualisation, resources, writing – review and editing, supervision.

## Conflicts of interest

There are no conflicts to declare.

## Supplementary Material
